# Media Induced Factitious Disorder by Proxy

**Published:** 2004

**Authors:** Prathama Guha, Om Prakash Singh, Moloy Ghosal

**Affiliations:** 1 RMO Cum Clinical Tutor, Dept. of Psychiatry, Burdwan Medical College & Hospital, West Bengal, India; 2 Asst. Prof & Head, Dept of Psychiatry, Burdwan Medical College & Hospital, West Bengal, India; 3 Asso. Professor, Dept of Psychiatry, Burdwan Medical College & Hospital, West Bengal, India

**Keywords:** Factitious disorder by proxy, FD, FD by proxy, Media, Internet

## Abstract

Perpetrators of Factitious Disorder by proxy are usually driven by motives such as garnering attention, mobilizing sympathy, acting out anger or controlling others. Widespread media coverage provides an opportunity for fulfilling all these needs. We describe a case of Factitious Disorder by proxy with a rather unusual ocular complaint. Circumstantial evidence indicates that the presentation may have been influenced by a similar case from the same locality in the preceding month, which received extensive media attention. The role of media on shaping psychopathology is discussed. Comparisons are drawn with other media influenced cases reported in the recently.


*Indian Journal of Psychiatry, 2004, 46(I)81-82*

### Media Induced Factitious Disorder by Proxy

**Prathama Guha*[1] Om Prakash Singh[2] Moloy Ghosal[3]**

**Abstracts**

Perpetrators of Factitious Disorder by proxy are usually driven by motives such as garnering attention, mobilizing sympathy, acting out anger or controlling others. Widespread media coverage provides an opportunity for fulfilling all these needs. We describe a case of Factitious Disorder by proxy with a rather unusual ocular complaint. Circumstantial evidence indicates that the presentation may have been influenced by a similar case from the same locality in the preceding month, which received extensive media attention. The role of media on shaping psychopathology is discussed. Comparisons are drawn with other media influenced cases reported in the recently.

Key words : Factitious disorder by proxy, FD, FD by proxy, Media, Internet.

### INTRODUCTION

Few patients are more challenging and troublesome to clinicians than those with factitious illnesses. The DSM – IV describes Factitious Disorder (FD) as an intentional production of symptoms with the sole motivation of assuming the sick role, in the absence of any external incentive.

Since the original description by Ashen (1951), numerous cases of FD have been documented. But the data on FD by proxy is still empirical and hence this condition is not a formal DSM – IV mental health diagnosis. Nonetheless, the research criteria for FD by proxy have been included in DSM-IV. This emphasizes the growing importance given to this variant of FD, first described by Meadow in 1977.

The perpetrators of the illness are typically mothers who induce signs and symptoms in their children. But exceptions do occur, as in this case, where an elder brother might have been responsible.

Patients with FD by proxy have intrigued clinicians in various specialities, including ophthalmology. In fact, patients with non organic visual complaints constitute upto 5% of a general opthalmologist's practice (Rajsekar et al., 1999). Most of these cases present with complaint of reduced visual acuity (Weller & Wiedemann, 1989). Others report diplopia, voluntary nystagmus, blepharospasm or psychogenic ptosis (Zahn, 1977; Cavenar et al., 1978, Newman, 1993). Conjunctival hypersensitivity, as in our case, is a relatively rare presentation (Walsh & Hoyf, 1969). Associated presence of insects in the lower fornix, to our knowledge, makes the case a first of its kind.

Searching for a plausible reason behind such an unusual presentation, we came across a striking case of 'urogenital myiasis' or the presence of fly maggots in the urogenital tract in a boy of similar age. He hailed from the same locality and presented in our hospital in the preceding month. That case had received widespread media attention. Within a month of this, the elder brother of our patient complained of finding ants in the child's left eye. Circumstantial evidence indicates that the desire for media coverage may have sparked off such an uncommon presentation.

### CASE REPORT

A 11 year old boy hailing from a village in West Bengal was brought by his family members to the ophthalmology OPD. The 'illness' began suddenly five days back when his elder brother, aged about 22 years discovered some dead ants in his left lower eye lid. Thereafter, his brother would find dead ants in his left eye everyday. The boy never found the ants himself, but occasionally felt minor irritation in his eye. The ants were a common variety in this part of Bengal (C. compressus), were never found alive and were always detected by the same person.

The unusual case caused quite a stir in their village. By the time the boy came to the hospital, his case had already been reported in a local newspaper.

There was no history of past medical or psychiatric illness. Family history was not contributory.

In view of no objective evidence of any ocular pathology, the case was referred to psychiatry OPD. On gentle questioning, the boy reported he had never found the ants himself, but were shown by his brother. He had no false belief about being infested by insects. He was, nonetheless, anxious for an explanation for his brother's findings. He reported no difficulty in vision and harboured no delusion about being seriously ill.

[1]RMO Cum Clinical Tutor, Dept. of Psychiatry, Burdwan Medical College & Hospital, West Bengal (VII-14 'Purbachal', Sector – 3), Salt Lake, Kolkata – 700 037.

[2]Asst. Prof & Head, Dept of Psychiatry, Burdwan Medical College & Hospital, West Bengal

[3]Asso. Professor, Dept of Psychiatry, Burdwan Medical College & Hospital, West Bengal.

*Correspondence

His brother had accompanied him to our OPD. He was very reluctant to let the boy get interviewed alone, and later charged doctors of having intimidated him.

Subsequently, the child's left eye was lightly bandaged. He stayed in the ward with his mother. Visitors were barred entry. With this arrangement, there was no further reporting of presence of ants or eye irritation. This management however, caused resentment among the family members. They felt the child and his mother were forced to underplay their complaints.

Detailed probing did not reveal any economic or material gain by the family on account of the illness. However, an interesting coincidence surfaced on probing. Just about a month back, a boy from the same village had reported passing of 'insects' in his urine. He had been admitted to the department of urosurgery in our hospital and treated as a rare case of urogenital myiasis. Both the print media and television, now almost ubiquitous in rural Bengal, showed considerable interest in the case, with interviews of the boy's family members being regularly published and telecasted.

The patient's family admitted knowledge about the previous case. But they vehemently rejected any suggestion of a relationship between the two cases.

Investigations done in the ophthalmology department included an ultrasonogram of the left eyeball and orbit, which revealed no abnormality. A diagnostic psychometry reported the child avoided response to most of the tests, giving only short, sketchy response to CAT and Rorschach Ink Blot Tests. Based on these, the psychologist was unable to make a definite diagnosis. Psychotherapy was suggested for the child and his family. But the family reacted angrily to the suggestion, and left the hospital. The case has not been available for follow-up.

### DISCUSSION

In the era of rapidly advancing communication technology, the importance of media in shaping human perceptions and even psychopathology is remarkable.

More recently, the internet, with its vast potential as a communication resource has received the attention of psychiatrists worldwide. Such widespread exposure made possible with minimal effort should come as a source of instant gratification for many patients, particularly those who harbour an intense need for attention. FD patients have frequently abused the Net. We cite a recent example of 4 cases, two of them assuming the role of terminal cystic fibrosis patients, one that of migraine, and one posing as a victim of extreme physical abuse (Feldman, 2000). All these patients misled. Net users for quite some time before being confronted for their inconsistencies.

The popular media and the Internet have also produced more organized psychopathology: Literature review yielded several case reports of 'Internet delusions' (Tan et al., 1997; Catalano et al., 1999; Catalano & Catalano, 2000; Podoll et al., 2000). Such delusions have been described to develop denovo in individuals without any psychiatric disorder (Catalano et al., 1999; Catalano & Catalano, 2000), and also in patients with chronic schizophrenia (Tan et al., 1997; Podoll et al., 2000; Duggal et al., 2002).

The psychodynamics underlying the behaviour of patients with Factitious disorder (or FD by proxy) may be the need to garner attention, underlying masochistic tendencies, a need to assume a dependant status, or to ease feelings of worthelessness. Equally powerful may be the desire to feel superior to authority figures that is gratified by deceiving the physician. The growing attention by the print and audiovisual media to 'unusual' clinical cases in a competitive communication market makes things easier for the patient, who is saved from the trouble of running from hospital to hospital for attention.

As the urban population moves towards the Internet and other advanced tools of communication, the psychology of the rural Indian is still swayed by the print media and television. This case reasserts the powerful influence of media on the average Indian psyche.

### REFERENCES

Catalano,G., Catalano M.C., Embi.C.S & Frankel, R.L. (1999) Delusions about the internet. Southern Medical Journal, 92, 609-610.

Catalano, G & Catalano, M.C. (2000) Social events & scientific innovations may affect content of delusions (reply). Southern Medical Journal, 93, 441.

Cavenar, J., rnatley, J.J. & Braasch, E(1978) Blepharorpasm: organic or functional? Psychosomatics, 19, 623-628

Duggal, H.S., Jagadheesan, K & Nizamie, S.H. (2002) 'Internet delusion' responsive to cognitive therapy . Indian Journal of Psychiatry, 44(3), 293-296.

Feldman, M.D. (2000) Manchausen by Internet: Detecting Factitious Illness and Crisis on the Internet. Southern Medical Journal. 93 (7) 669-672.

Newman, N.J. (1993). Clinical problems in psychiatric treatment of the medically ill: Neuro-opthalmology and Psychiatry. General Hospital Psychiatry, 15, 102-144

Podoll, K., Habermeyer, E., Noller, B., Elbel, H & Sass, H.(2000). The internet as a delusional topic in paranoid schizophrenia. Nervenartz, 71, 912 – 914.

Rajsekar, K., Rajsekar, Y.L & Chaturvedi S.K. (1999) Psycho-opthalmology: the interface between psychiatry and ophthalmology . Indian Journal of Psychiatry, 41(3) 186-196.

Tan, S., Shea, C, & Kopala, L. (1997) Paranoid Schizophrenia with delusions regarding the internet. Journal of Psychiatry and Neuro Science, 22, 143.

Walsh, F.B. & Hoyf, W.F. (1969) Clinical Neuro-ophthalmology.Edn.3, Vol.3 Baltimore: William & Wilkins, pp 2530.

Weller, F.B. & Wiedermann, P. (1989) Hysterical symptoms in ophthalmology Documents in Ophthalmology, 73, 1 – 33.

Zahn, J.R (1977) Incidence & characteristics of voluntary nystagmus. Archives of Ophthalmology, 95, 1399.

